# Supplementary material for: Imaging flow cytometry enables label-free cell sorting of morphological variants from populations of the unculturable bacterium Pasteuria ramosa
Source: mSphere. 2025 Nov 25;10(12):e01038-24. doi: 10.1128/msphere.01038-24 (PMC12724247; doi:10.1128/msphere.01038-24)
Supplement: Supplemental Material — Tables S1 to S4; Fig. S1 to S3. [file msphere.01038-24-s0001.pdf]

Supplementary Tables & Figures

	Imaging flow cytometer (Attune CytPix)		Cell Sorter (BD Influx)	
**Channel**	**Laser**	**Filter**	**Laser**	**Filter**
Ultraviolet-1 (UV1)			355	460/50
Ultraviolet-2 (UV2)				670/30
Violet-1 (V1)	405	450/50	405	460/50
Violet-2 (V2)		525/50		550/50
Violet-3 (V3)		610/20		
Violet-4 (V4)		660/20		
Violet-5 (V5)		710/50		
Violet-6 (V6)		780/60		
Blue-1 (B1)	488	530/30	488	530/40
Blue-2 (B2)		695/40		692/40
Blue-3 (B3)				610/20
Yellow-1 (Y1)	561	585/16	552	585/29
Yellow-2 (Y2)		620/15		610/20
Yellow-3 (Y3)		780/60		>750
Yellow-4 (Y4)				670/30
Red-1 (R1)	637	670/14	642	670/30
Red-2 (R2)		720/30		720/40
Red-3 (R3)		780/60		>750

Table S1. Detector configuration of the imaging flow cytometer and cell-sorter used in this study. Underlined text indicates that the two machines' parameters are identical. For each channel, the wavelength of light emitted after excitation with the laser light source and detected following separation with optical filters (center wavelength/bandwidth) is provided in nanometers. Equivalent channels are listed in the same row and given the same channel name in the main text.

	Used in cluster analysis	Not used in cluster analysis
**Scatter**	Forward scatter<br>Side scatter (width, height)	
**Fluorescence**	Pulse "height" (all channels)	Pulse area<br>Pulse width
**Image**<br>*"Texture"*	Intensity (average)<br>Intensity (SD)<br>Skewness intensity<br>Kurtosis intensity<br>Entropy intensity	Normalized intensity (average)<br>Normalized intensity (SD)<br>Normalized intensity (CV)<br>Intensity (CV)<br>Max. intensity<br>Min. intensity<br>Total Intensity
"Size & Shape"	Major diameter (µm)<br>Minor diameter (µm)<br>Area (μm^2)<br>Eccentricity (%)<br>Circularity (%)	Perimeter (µm)<br>Pseudodiameter (µm)<br>Minor to major axis ratio (%)
Other	Particle count	Pixel number

Table S2: Parameters acquired via imaging flow cytometry and used in the cluster analysis. Briefly, objects within images are identified via masking. The above parameters are then extracted. Intensity describes the "brightness" of each pixel of the masked object. The distribution of pixel-intensity within a particle is described by various parameters above e.g., skewness, kurtosis measures. For further details, see instrument documentation (37).

UMAP	Neighbors: 50,<br>Min. Low Distance: 0.00001,<br>Iterations: 500
FlowSOM	Random Cells, Linear, Gaussian, Euclidean,<br>Cluster centroids initialization method: random cells<br>Training decay function: Linear<br>2D Grid Neighborhood function: Gaussian<br>2D Grid Distance Metric: Euclidean<br>Coarse Training: 20 cycles<br>Fine-Tuning: 10 cycles, automatic neighborhood spread.

Table S3: Algorithm settings used in the pre-sort analysis. Settings used represent the defaults in FCS Express analysis software package.

	sorting					post-sort analysis	
	gate parameters (x|y)						
sample	1°	2°	3°	strategy id	no. sorted events	no. events validated	purity (% on-target)
**spores**							
a	R3|UV1 (1)	FSC|SSC (1)	-	s.A (1.1.1.)	24659	3000	85.3%
b	R3|UV1	FSC|SSC	-	s.A	100417	3000	97.9%
c	FSC|SSC (2)	UV1|UV2 (2)	-	s.B (2.2.1.)	416880	500	90.8%
f	FSC|SSC	R1|UV1 (5)	-	s.C (2.5.1.)	198207	500	93.0%
i	FSC|SSC	R1|UV1	-	s.C	34142	500	98.4%
j	FSC|SSC	UV1|FSC (8)	-	s.D (2.8.1.)	29662	500	89.2%
k	FSC|SSC	UV1|FSC	-	s.D	32657	500	97.6%
l	FSC|SSC	UV1|FSC	-	s.D	111339	500	96.8%
n	FSC|SSC	UV1|FSC	R3|V1 (9)	s.E (2.8.9.)	20573	500	98.4%
o	FSC|SSC	UV1|FSC	R3|V1	s.E	23099	500	97.2%
p	FSC|SSC	UV1|FSC	R3|V1	s.E	85809	500	98.0%
q	FSC|SSC	UV1|FSC	R3|V1	s.E	3680	500	76.4%
r	FSC|SSC	UV1|FSC	R3|V1	s.E	1873	157	81.5%
t	FSC|SSC	UV1|FSC	R3|V1	s.E	318508	500	99.0%
u	FSC|SSC	UV1|FSC	R3|V1	s.E	50410	500	98.2%
**cauliflower**							
d	FSC|R3 (3)	FSC|UV1 (3)	R3|FSC (2)	c.A (3.3.2.)	5064	601	95.5%
k	FSC|R3	UV1|SSC (9)	-	c.B (3.9.1.)	4073	448	87.3%
l	FSC|R3	UV1|SSC	SSC-W|SSC (6)	c.C (3.9.6.)	11228	500	74.8%
m	FSC|R3	UV1|SSC	R3|UV1 (7)	c.D (3.9.7.)	2169	500	79.6%
n	FSC|R3	UV1|SSC	R3|UV1	c.D	1878	500	92.8%
o	FSC|R3	UV1|SSC	R3|UV1	c.D	1485	500	88.0%
q	FSC|R3	UV1|SSC	R3|UV1	c.D	6972	500	89.8%
r	FSC|R3	UV1|SSC	R3|UV1	c.D	10643	500	97.0%
s	FSC|R3	UV1|SSC	R3|UV1	c.D	10850	500	96.8%
**grapes**							
a	R3|UV1	FSC|SSC	-	g.A (1.1.1.)	4752	2457	91.0%
b	R3|UV1	FSC|SSC	-	g.A	20945	3000	93.5%
e	FSC|SSC	FSC|UV1	SSC|R1 (3)	g.C (2.3.3.)	3856	500	88.0%
f	FSC|SSC	UV1|R3 (4)	FSC|R1 (4)	g.D (2.4.4.)	35521	500	93.8%
g	FSC|SSC	UV1|R3	FSC|R1	g.D	157471	500	95.4%
h	FSC|SSC	UV1|Y2 (6)	UV1|R3 (5)	g.F (2.6.5.)	16233	500	93.0%
i	FSC|SSC	UV1|Y2	-	g.E (2.6.1.)	5194	706	94.6%
j	FSC|SSC	UV1|B2 (7)	-	g.G (2.7.1.)	18955	500	83.4%
k	FSC|SSC	UV1|B2	-	g.G	16741	500	91.2%
l	FSC|SSC	UV1|B2	-	g.G	213551	500	76.8%
n	FSC|SSC	UV1|SSC-W (10)	UV1|B2 (8)	g.B (2.10.8.)	10629	500	99.2%
o	FSC|SSC	UV1|SSC-W	UV1|B2	g.B	20132	500	92.2%
p	FSC|SSC	UV1|SSC-W	UV1|B2	g.B	34310	500	96.4%
**activated**							
a	R3|UV1	FSC|SSC	-	a.A (1.1.1.)	9191	3000	77.0%
c	FSC|SSC	UV1|UV2	-	a.B (2.2.1.)	2541749	500	75.0%
o	FSC|R3	UV1|SSC-W	-	a.C (3.10.1.)	967	472	92.2%
p	FSC|R3	UV1|SSC-W	-	a.C	1048	500	95.4%
t	FSC|SSC	UV1|UV2	-	a.B	192726	500	99.0%
u	FSC|SSC	UV1|UV2	-	a.B	89568	500	96.6%

Table S4: Gating parameters for each sort Samples (1st column) were arbitrarily given an alphanumeric name (a-x), in ascending order of processing. Note that each sample contained 1 or more morphotypes (e.g., note that sample "n" comprised sufficient spores, cauliflowers and grapes). Each row represents an individual sort. Parameters used to define the axes (x parameter | y parameter) of the primary (1°), secondary (2°) and tertiary (3°) sorting gates are given; each unique parameter combination is given a numeric id within parentheses upon its first appearance in each column. For each target morphotype, each unique gating strategy is assigned an id (4th column); to enable comparison among sorting strategies of different morphotypes the id of the primary, secondary and tertiary gate-parameters are listed in parentheses. Of the total sorted events (no. sorted events, 6th column), a subset were imaged via IFC (no. events validated, 7th column) yielding an assessment of the purity (% on-target, 8th column) of each sort.

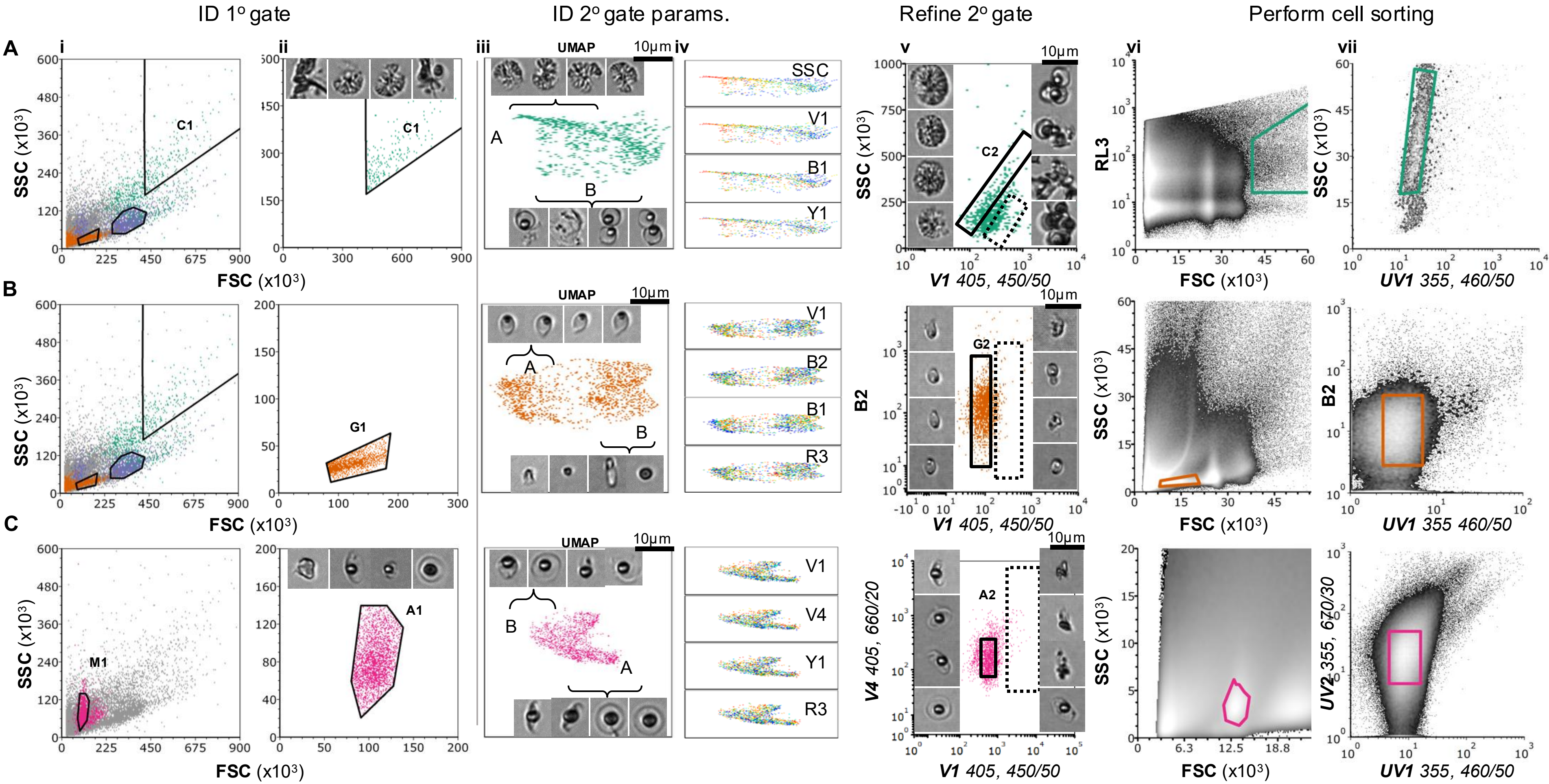

Figure S1: Using IFC to design a strategy to sort diverse morphotypes of *Pasteuria*. Worked example of the procedure used to cell sort cauliflower (A, green), grape (B, orange) and activated stages (C, pink) of *Pasteuria.* The analysis presented in A, B (and Fig 1) was performed using the same single sample; that presented in C used a different sample. Briefly, as described in the main text (Fig. 1A-C), we (i, ii) identified a primary (1°) gate that separated each of our target morphotypes from one another (here FSC-SSC) using the results of the cluster analysis. We then (iii) identified regions of each target cluster that contained a “pure” vs. mixed population of our target morphotype (A, B, respectively) using the image data. By (iv) coloring the clusters by each fluorescence parameter, we identified secondary parameters that could be used to discriminate target morphotypes from debris. Finally, (v) the position of the secondary gate (solid box) was set with respect to the image data (v, surrounds; images of in-gate events left, out-of-gate images right). We then performed cell sorting. Here, as in Fig. 1, the parameters used for sorting differed in a minor way from those identified via IFC (compare Ai vs. Avi; Bv vs. Bvii; Cv vs. vii); we indicate these differences in italicized text and provide laser/filter values for each of the relevant parameters. We deviated from the IFC-identified sorting strategy for one of two reasons. First, if it was possible to readily identify the target morphotype using a higher-order gate, we avoided setting additional gates. For example, here (A) we could readily distinguish cauliflower stages via their high FSC signal even using the R3-FSC gate, which is used to exclude algae in the pre-gating analysis step (see Methods). Alternatively, we amended the gating strategy when we found that events were better distinguishable using a parameter that was available on the sorter, but not on the imaging cytometer e.g., UV1 better distinguished spores, grapes and activated stages than V1 (B,C; Fig. S3). Scale bar above panel iii. also apply to panel ii, in the same row.

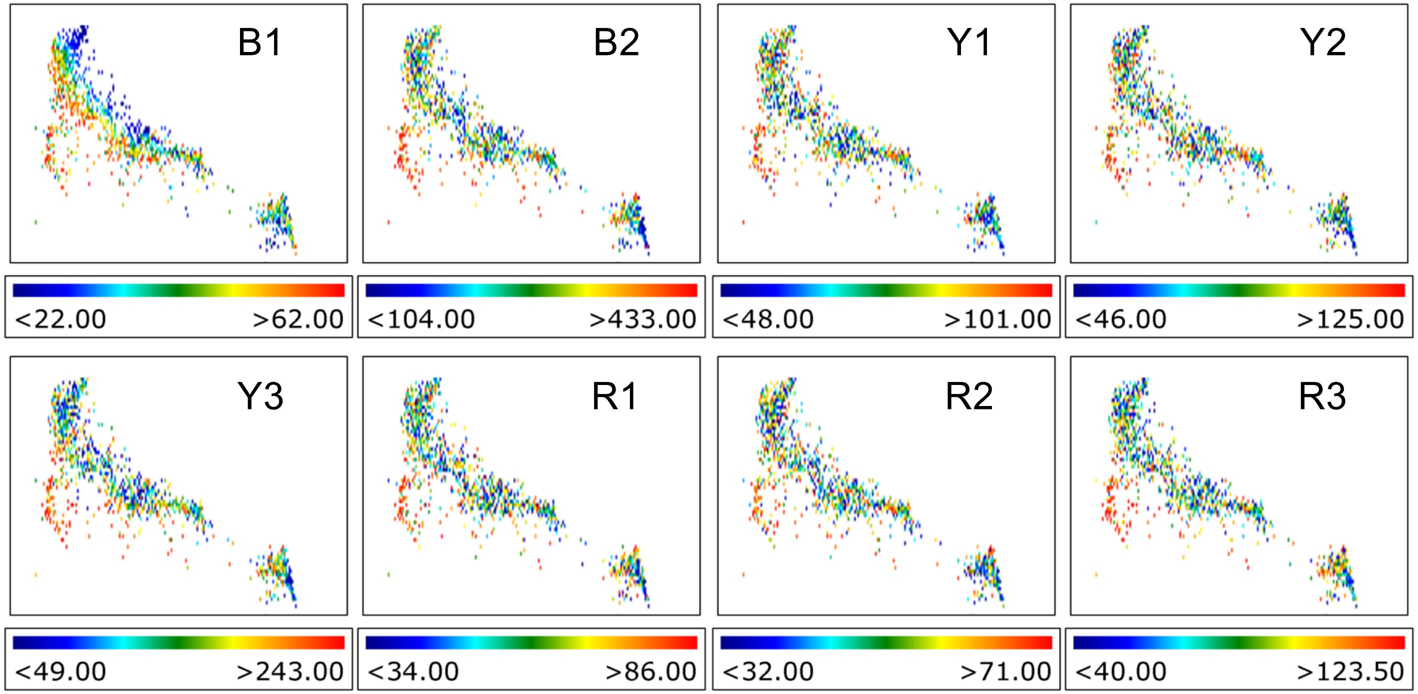

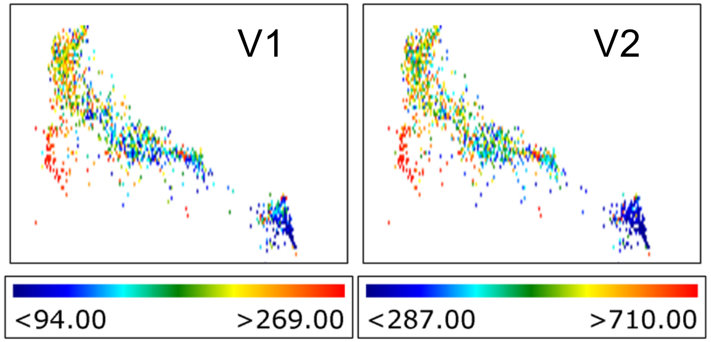

Figure S2: UMAP analysis reveals autofluorescent signatures that can be exploited in secondary gating of *Pasteuria* spores. Events presented in Cluster 3 in Fig. 1 of the main text, colored by the intensity of fluorescence in the 10 channels common to the sorter and cytometer (see Table S1). Each parameter was rescaled linearly so that it contained only the range of values emitted by events in the clusters; colors indicate the % intensity of each event, relative to the scale.

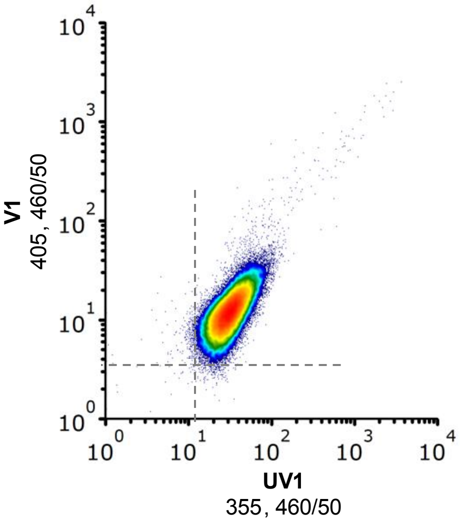

Figure S3: ***Pasteuria*** **spores autofluorescence with higher intensity in the UV1 than V1 channel.** Density of events in gate S1 (primary spore gate) in UV1-V1 space, as analyzed on the sorter (high density, warm colors; low density, cool colors). Dashed line imposed on the plot to enhance readability.
